# Molecular and Macromolecular Interactions of Carbon-Based Nanostructures

**DOI:** 10.3390/ijms24010619

**Published:** 2022-12-30

**Authors:** Valentina Villari

**Affiliations:** CNR—Istituto per i Processi Chimico-Fisici, Viale F. Stagno d’Alcontres 37, 98158 Messina, Italy; villari@ipcf.cnr.it

The interactions of molecules and macromolecules with carbon nanostructures such as carbon dots, carbon nanotubes, graphene, graphene oxide, and fullerenes, have been stimulating the interest of the researchers working on the preparation, functionalization, properties and applications of carbon-based nanomaterials. Carbon nanostructures, indeed, display very interesting electronic conductivity, high mechanical strength, low density and easiness in chemical functionalization, by adopting either covalent or supramolecular approaches, to fine-tune the properties of the resulting nanocomposite materials for specific aims. In addition, the variety of size, shape, and dimensionality makes these structures particularly versatile in many fields: beyond their promising prospects for a new generation of solar cells, and light sensitive elements and electronics [1,2,3,4,5,6], more recently the attention has been turned onto industrial, environmental, and biomedical aspects [7,8,9].

A huge amount of complex research and both theoretical and experimental studies have been performed in this regard, so that this special issue does not aim at being exhaustive but rather at giving some spotlights on carbon nanostructures with properties useful for facing the issues related to their applications and seeing them within the context of some recent literature.

Certainly, the studies on carbon nanostructures for photovoltaic and energy industry have experienced a tremendous growth in the last two decades because of the need to reach the next Sustainable Development Agenda targets. For example, they can be realistically employed in various elements of a solar cell, as well as their composites, but the number of concrete applications is still rather limited because of the high cost in obtaining pure materials and of the current better performance of silicon-based materials in the visible region. Furthermore, dispersibility in water seems to be a requirement for having access to better yields and lower production costs, so that the typical hydrophobic character of some carbon nanostructures has to be overcome by proper functionalization.

However, the dispersibility in aqueous medium is imperative for other application-oriented studies like those involving medical sciences and healthcare industry, and carbon nanostructures can be functionalized and surface-modified with a variety of molecules to adapt their properties for drug delivery, cell imaging, biosensors, and other biomedical devices [7,10]. Fullerenes derivatives and carbon nanotubes composites are promising for anticancer therapies, gene delivery, and are also proposed as radio-protectant for human erythrocytes against ionizing radiation damage [11]. Some carbon-based nanoparticles have been reported to possess bactericidal properties and inhibition of viruses [12], whereas graphene and carbon nanotubes can be applied in rapid diagnostic kits for COVID-19 [13,14], entrapped in matrices for realizing personal protective equipment, or embedded in polymer substrates as devices for artificial synapses in neural networks [15]. Additionally, tissue engineering, especially concerning bone tissues, is open to the new materials based on carbon nanostructures which offer high elastic modulus, the possibility to form hard composite scaffolds similar to bone structure and to be incorporated in biocompatible polymer matrices in order to minimize cytotoxicity [16].

Toxicity and immunogenicity of carbon-based nanomaterials [17,18,19,20] represent a reasonable concern to take in serious account not only for biomedical applications. Indeed, the nanotechnology based on these materials and their composites offers effective solutions to the agriculture industry to increase crop yield and protect plants, to the food packaging industry aiming at improving conservation and prolonging the shelf-life of the food products [21], to the plastic industry to increase tensile strength, and to the methods for detection and filtering of pollutants (from volatile organic compounds and heavy metals to pesticides). All of these solutions imply the use of huge amount of carbon-based nanomaterials for the large-scale production which, even with low toxicity, can expose the environment, the biological organisms and hence the human health to the risk related to accumulation [22,23].

Therefore, along with the study focused at overcoming the current limitations of carbon-based nanomaterials and at improving their performance for applications, also many investigations are recently concerning the understanding of their structure-related interactions with molecules in biological processes and the development and implementation of green routes for their sustainable production [24,25] and recycling [26].

To conclude, the current literature shows that the technological prospects of carbon nanostructures for the future are manifold and that, if the technological approaches will proceed at the same rate as the adoption of greener routes and more systematic studies of their toxic effects and bio-safety, these innovative materials could represent a concrete benefit of society and environment. The Editors’ wish is that this special issue, by dealing with some important topics related to the realization, study, application, potentiality, drawbacks, and problems of carbon-based nanomaterials, can contribute to stimulate new investigation hints towards this target.
